# Human neutrophil peptide-1 promotes alcohol-induced hepatic fibrosis and hepatocyte apoptosis

**DOI:** 10.1371/journal.pone.0174913

**Published:** 2017-04-12

**Authors:** Rie Ibusuki, Hirofumi Uto, Kohei Oda, Akihiko Ohshige, Kazuaki Tabu, Seiichi Mawatari, Kotaro Kumagai, Shuji Kanmura, Tsutomu Tamai, Akihiro Moriuchi, Hirohito Tsubouchi, Akio Ido

**Affiliations:** 1 Digestive and Lifestyle Diseases, Department of Human and Environmental Sciences, Kagoshima University Graduate School of Medical and Dental Sciences, Kagoshima, Japan; 2 Department of International Island and Community Medicine, Kagoshima University Graduate School of Medical and Dental Science, Kagoshima, Japan; 3 Center for Digestive and Liver Diseases, Miyazaki Medical Center Hospital, Miyazaki, Japan; 4 Department of HGF Tissue Repair and Regenerative Medicine, Kagoshima University Graduate School of Medical and Dental Sciences, Kagoshima, Japan; 5 Kagoshima City Hospital, Kagoshima, Japan; University of Navarra School of Medicine and Center for Applied Medical Research (CIMA), SPAIN

## Abstract

**Background and aims:**

Neutrophil infiltration of the liver is a typical feature of alcoholic liver injury. Human neutrophil peptide (HNP)-1 is an antimicrobial peptide secreted by neutrophils. The aim of this study was to determine if HNP-1 affects ethanol-induced liver injury and to examine the mechanism of liver injury induced by HNP-1.

**Methods:**

Transgenic (TG) mice expressing HNP-1 under the control of a β-actin-based promoter were established. Ethanol was orally administered to HNP-1 TG or wild-type C57BL/6N (WT) mice. SK-Hep1 hepatocellular carcinoma cells were used to investigate the effect of HNP-1 on hepatocytes *in vitro*.

**Results:**

After 24 weeks of ethanol intake, hepatic fibrosis and hepatocyte apoptosis were significantly more severe in TG mice than in WT mice. Levels of CD14, TLR4, and IL-6 in liver tissues were higher in TG mice than in WT mice. Apoptosis was accompanied by higher protein levels of caspase-3, caspase-8, and cleaved PARP in liver tissue. In addition, phosphorylated ASK1, ASK1, phosphorylated JNK, JNK1, JNK2, Bax, Bak and Bim were all more abundant in TG mice than in WT mice. In contrast, the level of anti-apoptotic Bcl2 in the liver was significantly lower in TG mice than in WT mice. Analysis of microRNAs in liver tissue showed that miR-34a-5p expression was significantly higher in TG mice than in WT mice. Furthermore, in the presence of ethanol, HNP-1 increased the apoptosis with the decreased level of Bcl2 in a concentration-dependent manner *in vitro*.

**Conclusions:**

HNP-1 secreted by neutrophils may exacerbate alcohol-induced hepatic fibrosis and hepatocyte apoptosis with a decrease in Bcl2 expression and an increase in miR-34a-5p expression.

## Introduction

Alcoholic liver disease (ALD) is a well-known disease that can progress from simple steatosis to liver cirrhosis [1]. Currently, the incidence and mortality of liver diseases are high worldwide [2,3], and alcohol consumption significantly affects the pathology of chronic liver diseases [2,3]. Mortality related to alcohol increased from 1990 to 2010, and alcohol accounted for 2.8% of all deaths worldwide in 2010 and for 47.9% of deaths from liver cirrhosis [3]. Thus, ALD is a significant risk factor for liver cirrhosis and hepatocellular carcinoma, and a major cause of death [4,5]. However, the treatment protocol for ALD is not well established. Liver damage in ALD is caused by excess alcohol consumption and generally the disease develops in individuals who drink ≥60 g/day of ethanol for ≥5 years [4]. More than 95% of patients with ALD develop hepatic steatosis and 20–40% develop alcoholic steatohepatitis (ASH), which subsequently progresses to hepatic fibrosis, liver cirrhosis, and hepatocellular carcinoma [4,5]. The pathology of ALD varies with gender, age, nutritional status, genetic predisposition, smoking status, and pattern of drinking, and the mechanism of pathological progression is uncertain [4,5].

Increases in serum alanine aminotransferase (ALT) and aspartate aminotransferase (AST) are simple indices of liver damage. ALT and AST are widely used for screening, diagnosis, and prediction of prognosis in patients with liver diseases, and serve as an index of hepatocellular death [4,5]. This is a common event not only in hepatitis B virus (HBV) [6] and hepatitis C virus (HCV) [7] infections, but also in alcoholic steatohepatitis (ASH) [4,8] and nonalcoholic steatohepatitis (NASH) [9], and is caused by necrosis or apoptosis. Hepatocyte apoptosis is a biochemically regulated process characterized by DNA fragmentation [10]. Apoptosis of hepatocytes containing Mallory bodies occurs in ALD, and activation of caspase induces significantly greater hepatocyte apoptosis as a key trigger in pathological progression of ALD [8].

Neutrophil infiltration in the liver is a histological characteristic of alcoholic hepatitis (AH) [4,5,8,11,12] and similar histopathological changes are observed in rodent models [13]. Hepatocyte apoptosis is also induced by neutrophil infiltration in patients with ASH [11,12]. Specifically, neutrophils infiltrate the hepatic parenchyma, and neutrophil-derived reactive oxygen species (ROS) and proteases damage hepatocytes [14]. Neutrophil infiltration is associated with the severity of AH and neutrophils are involved in alcoholic liver injury [5,11–14]. However, the roles of neutrophils in development of alcoholic liver injury have not been fully determined.

Defensins produced by human neutrophils are classified into α- and β-defensins. The α-defensins include human neutrophil peptide-1 (HNP-1), which is stored in azurophil granules of neutrophils [15]; HNP-2, -3 and -4 which are mainly secreted by neutrophils, along with HNP-1; and human defensin (HD)-5 and -6, which are found in Paneth cells and other cells in the small intestine [16]. The sequences of HNP-1 and -3 are identical except for one amino acid at the 3' end, and the functions of these peptides appear to be the same [17]. In a serum proteomics study of patients with ulcerative colitis (UC) indicated for leukapheresis, we found that HNP-1 is a novel biomarker for the pathology of UC [18] and a possible factor involved in aggravation of the disease [19]. Infiltration of neutrophils and polymorphonuclear leukocytes in the liver is a histological characteristic of ALD and NASH [20], and leukapheresis may be an effective therapy for severe alcoholic hepatitis [21]. Ludwig also suggested that the pathologies of NASH and ALD are similar and involve mitochondrial disorder, oxidative stress, endotoxin, and TNF-α [22]. We have also shown that HNP-1 enhances hepatic fibrosis in a NASH mouse model fed a choline-deficient L-amino acid-defined (CDAA) diet [23].

Based on these findings, we hypothesized that HNP-1 promotes aggravation of alcoholic hepatitis. In this study, we show that HNP-1 does not influence alcoholic steatohepatitis, but enhances hepatic fibrosis and hepatocyte apoptosis. We also show that HNP-1 upregulates expression of a microRNA that is known to inhibit expression of the anti-apoptotic factor B-cell lymphoma 2 (Bcl2). These findings provide new insights into the mechanism of progression of ALD and may facilitate development of treatment for ALD.

## Material and methods

### Animal experiments

The CAG promoter was used to generate HNP-1 transgenic mice expressing HNP-1 cDNA [23]. There is no rodent model of ALD that replicates human ALD with progression to fibrosis or cirrhosis without addition of a secondary insult [24], but we speculated that long-term alcohol feeding may lead to development of mild fibrotic deposition and may increase inflammatory cell infiltrates. Therefore, groups of mice were allowed to ingest 10% ethanol freely for 8 or 24 weeks: an 8-week intake group (7 HNP-1 TG [TG] mice and 6 wild-type [WT] mice) and a 24-week intake group (11 TG mice and 6 WT mice). Mice were sacrificed after 8 or 24 weeks of ethanol intake. Blood was collected by cardiopuncture and the liver was preserved. All mice were male, and TG and WT mice were aged 9–10 and 9 weeks, respectively. The experimental protocols used in this research were approved by the ethical committee of Kagoshima University (Permit Numbers: MD13024).

### Biochemical measurements

ALT, lactate dehydrogenase (LDH), glucose, triglycerides, and total cholesterol in serum were determined by SRL (Tokyo, Japan). The level of serum HNP-1 was determined using a HNP1-3, Human, ELISA kit (Hycult Biotech). Liver NFκ-B activity was determined using a TransAM^®^ NFκ-B family ELISA Kit (Active Motif).

### Histological study

Liver tissue was immersed in 10% formalin and fixed with paraffin. Two tissue sections per liver were stained with hematoxylin-eosin (HE) to evaluate the severity of inflammation. Neutrophils were counted in 4 randomly selected high-power fields (HPF = 0.09766 mm^2^ at magnification 400×) and expressed as number of cells per square millimeter of liver surface [25]. Hepatic fibrosis was evaluated by Sirius Red, AZAN, and α-smooth muscle actin (α-SMA) staining. Immunostaining was performed using antibodies against α-SMA (Millipore, Billerica, MA), F4/80, CD68 (both from AbD Serotec, Raleigh, NC), and phospho-NF-κBp65 (Cell Signaling, Beverly, MA). Apoptosis was evaluated by TUNEL staining using an apoptosis detection kit (DeadEnd TUNEL, Promega, Madison, WI). Six spots were randomly selected from each stained section, and apoptosis was evaluated in 12 fields per liver. Images were taken at 40× magnification and the positive area was evaluated using a QuickGrain digital image analyzer (Inotech, Hiroshima, Japan). The severity of hepatic steatosis was evaluated by imaging of lyophilized liver tissue stained by oil red-O and the triglyceride level in liver tissues as previously described [23].

### RNA isolation, reverse transcription, and real time quantitative polymerase chain reaction

Total RNA was isolated from liver tissue stored at -80°C using Trizol reagent (Invitrogen) and a PureLink^®^ RNA Mini Kit. cDNA was synthesized from 0.5 μg of total RNA using a Prime Script RT Reagent Kit (Takara). Then, real-time polymerase chain reaction (PCR) with SYBR^®^ Green I intercalation was performed using a StepOnePlus™ Real-time PCR System (Applied Biosystems). Gene expression levels were analyzed using the ΔΔCt method, with the level of each gene was corrected based on that of Glyceraldehyde 3-phosphate dehydrogenase (GAPDH). PCR primer sequences are shown in S1 Table.

### Western blot analysis

Protein extraction from liver tissue and cells was performed using T-PER and M-PER tissue protein extraction reagents (Thermo Fisher Scientific Inc.), respectively. Protein levels were determined using a DC™ (detergent compatible) protein assay kit (Bio Rad) to adjust to 1 μg/μl. The primary antibodies used in the study were those against type 1 collagen, catalase (both from Millipore), α-SMA (Abcam, Cambridge, UK), CD14 (Santa Cruz Biotechnology Inc, Santa Cruz, CA), toll-like receptor (TLR)-4, phosphorylated NFκ-Bp65 (p-NFκ-Bp65), NFκ-Bp65, interleukin (IL)-6, caspase 3, caspase 8, cleaved- poly (ADP-ribose) polymerase (PARP), apoptosis signal-regulating kinase (ASK)1, phosphorylated ASK1 (p-ASK1), c-Jun N-terminal kinases (JNK)1, JNK2, phosphorylated JNK (p-JNK), Bid, Bak, Bax, Bim, Bcl2, protein kinase-like endoplasmic reticulum kinase(PERK), CCAAT-enhancer-binding protein homologous protein (CHOP), and p53 upregulated modulator of apoptosis (PUMA), β-actin, (all from Cell Signaling). Anti-rabbit-horseradish peroxidase (HRP) and anti-mouse-HRP (both from Santa Cruz Biotechnology) were used as secondary antibodies. The ECL (ECL Prime Western Blotting Detection Reagents, GE) reaction was developed using a Fluor Chem FC2 Imaging System (Alpha Innotec) and quantified with Image J Software.

### MicroRNA analysis

#### MicroRNA extraction and reverse transcription

miRNAs from mouse liver tissues and human cells were extracted and purified using a miRNeasy Mini Kit (Qiagen, 217004). RNA was eluted in 30 μL of RNase-free water and stored at -80°C. Reverse transcription was performed using a miScript II RT Kit (Qiagen, 218161). The resulting cDNA was used for quantitative real-time PCR.

#### Real-time PCR using a pathway-focused miScript miRNA PCR array

miRNA expression was evaluated using a 96 well-plate miScript miRNA PCR array (Qiagen, MIMM-114Z), which contains miScript primers for 84 well-characterized miRNAs and duplicates of 6 internal reference miRNAs. After an initial incubation step of 15 min at 95°C, the PCR thermo-cycle consisted of denaturing at 94° for 15 s, annealing at 55° for 30 s, and extension at 70° for 30 s. Relative quantification was performed by the ΔΔCT method.

#### Detection of microRNA 34a (miR34a-5p) by real-time PCR

Expression of miRNA 34a-5p was analyzed using a target-specific miScript primer assay (forward primers) and the miScript SYBR Green PCR Kit (Qiagen 218073), which contains the miScript Universal Primer (reverse primer) and QuantiTect SYBR Green PCR Master Mix. The miScript PCR Control primer is RNU6-2 and the target primer is mmu-miR-34a-5p (MIMAT0000542).

### Cell culture

SK-Hep1 human hepatocellular carcinoma cells (European Collection of Cell Culture) were used. Cells were cultured in Dulbecco's Modified Eagle's Medium (DMEM) containing 10% fetal bovine serum (FBS) and 1% penicillin in 5% CO_2_ at 37°C. Recombinant HNP-1 was obtained from the Peptide Institute (Osaka, Japan) [23].

#### Caspase-3/7 detection

Caspase-3/7 activity was evaluated using CellEvent™ Caspase-3/7 Green Detection Reagent (Life Technology). SK-Hep1 cells were adjusted to 1×10^5^/ml and cultured in a 6-well plate for 24 h. After removal of supernatant, 3 ml/well each of recombinant HNP-1 (0, 10, 40, and 100 ng/ml) was added in DMEM and the cells were incubated for 6 h at 37°C in 5% CO_2_. CellEvent™ Caspase-3/7 Green Detection Reagent was then added to a final concentration of 5 μM and incubated for 30 min at 37°C in 5% CO_2_. Caspase 3/7 activity was evaluated by fluorescence microscopy.

#### Detection of cell death

SK-Hep1 cells were cultured at a density of 1×10^4^/100 μl/well (96-well plate) for 24 h. To evaluate the influence of ethanol, 100 μl/well of DMEM containing ethanol (0, 10, 50, 100, 200 mM) was added to the SK-Hep1 cells after removal of supernatant and the cells were incubated for 6 h at 37°C in 5% CO_2_. Next, to evaluate the influence of HNP-1,100 μl/well of DMEM containing recombinant HNP-1 (0, 10, 40, 100 ng/ml) was added and the cells were incubated for 6 h to evaluate the influence of HNP-1. To study the influence of HNP-1 in the presence of ethanol, 100 μl/well of DMEM containing recombinant HNP-1 (0, 10, 40, 100 ng/ml) was added and the cells were incubated for 1 h and the supernatant was removed. Then, 100 μl/well of DMEM containing recombinant HNP-1 (0, 10, 40, 100 ng/ml) was added to DMEM medium containing 100 mM ethanol [26], and the cells were incubated for 5 h. Cell death was evaluated using a Cell Death Detection ELISA Plus (Roche Molecular Biochemicals).

### Statistical analysis

All data are expressed as the means ± standard deviations. In the animal studies, the differences between groups were analyzed using the non-parametric Man-Whitney *U*-test. In vitro studies, results are analysis by Tukey test. Statistical analysis was performed using IBM SPSS Statistics 20 (IBM Japan, Tokyo, Japan). The significance level was set at *P* < 0.05.

## Results

### Transgenic expression of HNP-1 increases liver weight gain and exacerbates liver injury in mice

In TG mice, serum HNP-1 levels were 33.8±11.5 (n = 7) and 56.5±16.4 (n = 11) in an 8-week ethanol intake groups and a 24-week ethanol intake groups, respectively. In contrast, HNP-1 was not detected in serum of WT mice with or without ethanol intake. There was no difference in liver to body weight ratio after 8-week ethanol intake between TG and WT mice (4.8±0.7 vs. 5.0±0.3, P = 0.70). In contrast, this ratio was significantly higher in the TG mice compared with WT mice after 24-week ethanol intake, although there was no difference in body weight between these mice (S2 Table). Biochemical data did not differ significantly between TG and WT mice without ethanol intake (data not shown). Serum ALT levels after 8-week ethanol intake in TG mice was similar to that in WT mice (55.3±17.3 vs. 51.3±11.0, P = 0.64). In contrast, ALT after 24-week ethanol intake was significantly greater in TG mice than in WT mice (S2 Table).

### Effect of HNP-1 on inflammatory cell infiltration and hepatic steatosis induced by ethanol in vivo

Alcoholic liver injury is characterized by infiltration of polymorphonuclear leukocytes and inflammatory cells [4,5,11–14,20]. Histological findings did not differ between TG and WT mice without ethanol intake (S1 Fig), but infiltration of inflammatory cells including neutrophils in the liver in TG mice was higher than that in WT mice in HE-stained liver tissue after 8- and 24-week ethanol intake (S1 Fig). In addition, nuclear enlargement and cell degeneration of hepatocytes were more common in TG mice compared with WT mice in HE-stained liver tissue after 24-week ethanol intake (S1 Fig). In contrast, comparison of the severity of hepatic steatosis by oil red-O staining indicated no difference in fatty changes in the liver between TG and WT mice after 24-week ethanol intake (S2A Fig). There was also no difference in the amount of triglycerides in the liver between TG and WT mice after 8- and 24-week ethanol intake (S2B Fig).

### Transgenic expression of HNP-1 enhances liver fibrosis

**Fig 1:**
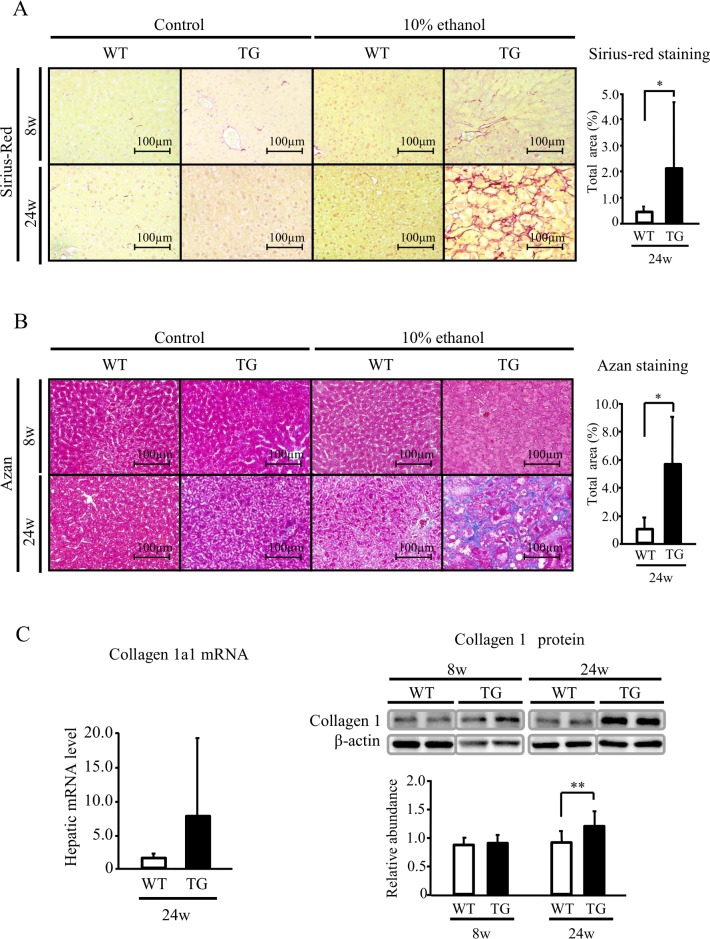
Hepatic fibrosis assessed by Sirius red and Azan staining, and collagen 1 expression. (A) Sirius red staining. (B) AZAN staining. (C) Expression of collagen 1a1 mRNA and type 1 collagen protein in liver tissue. Results are shown as means ± SD (7 TG mice and 6 WT mice in the 8-week intake group, and 11 TG mice and 6 WT mice in the 24-week intake group). WT, wild type; TG, HNP-1 transgenic mice. *P = 0.*001*, **P<0.*05*

**Fig 2:**
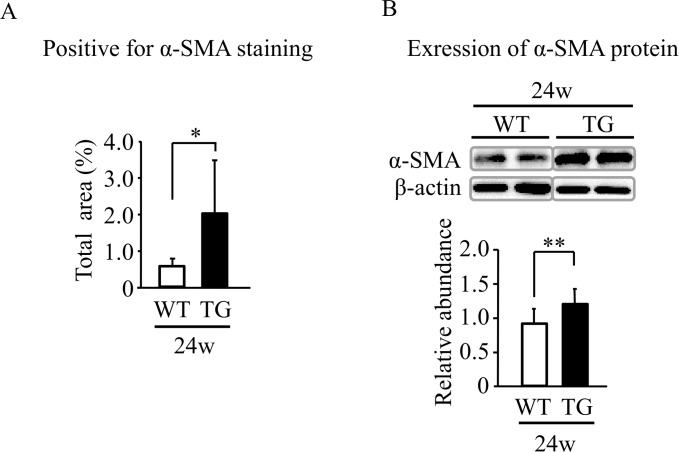
Expression of α-smooth muscle actin in liver tissue. (A) Semi-quantification for positive area of immunostaining using anti-α-smooth muscle actin antibody in the 24-week model. (B) α-smooth muscle actin expression assessed by Western blot analysis. Results are shown as means ± SD (*n* = 11 in TG mice and *n* = 6 in WT mice). SMA, smooth muscle actin. *P = 0.*001*, **P<0.*05*

There was no difference in hepatic fibrosis between TG and WT mice after 8-week ethanol intake. In contrast, after 24-week ethanol intake, hepatic fibrosis was significantly greater in TG mice than in WT mice (Fig 1A and 1B). The levels of collagen1A1 mRNA and collagen1 protein in liver tissue were also significantly greater in TG mice than in WT mice after 24-week intake (Fig 1C). These findings suggest that HNP-1 might promote hepatic fibrosis in mice with ALD. In addition, after 24-week ethanol intake, there were significantly more α-SMA-positive cells in liver tissue and a significant increase in α-SMA protein, an index of activation of hepatic stellate cells, in TG mice (Fig 2A and 2B and S3 Fig). These results indicate that HNP-1 further activated hepatic stellate cells that were activated by 24-week ethanol intake.

### Greater macrophage infiltration and CD14 and TLR4 levels in HNP-1 TG mice with ethanol intake

**Fig 3:**
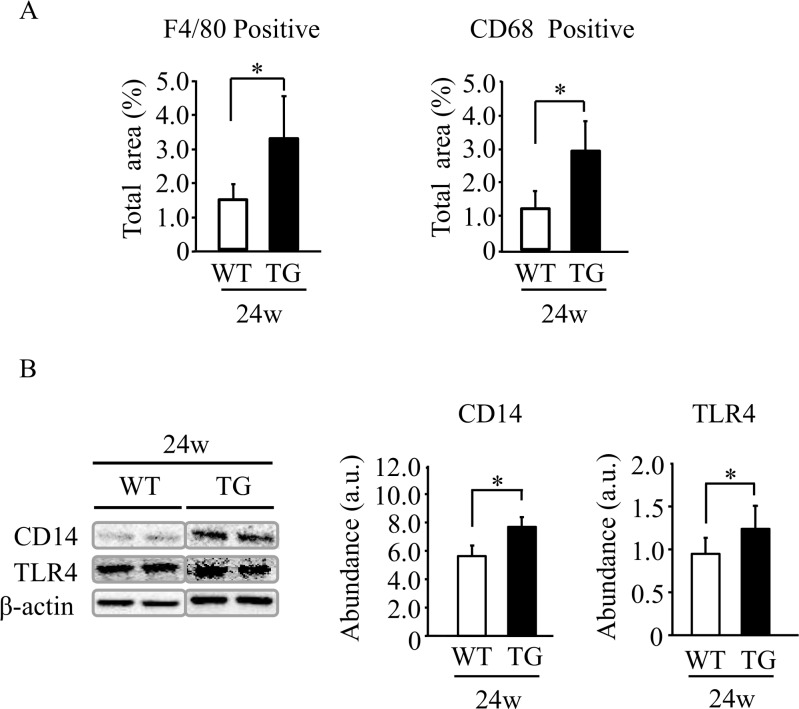
Expression of F4/80, CD68, CD14 and toll-like receptor 4 in liver tissue. (A) Semi-quantification of positive cells for immunostaining using anti-F4/80 or anti-CD68 antibody. (B) Semi-quantification of CD14 and toll-like receptor 4 protein expression assessed by Western blot analysis. Results are shown as means ± SD (*n* = 11 in TG mice and *n* = 6 in WT mice). *P<0.*05*.

Kupffer cells are hepatic macrophages that play an important role in development of alcoholic liver injury [4,5,27]. The levels of F4/80-positive and CD68-positive macrophages (S4 Fig and Fig 3A) and of proteins such as CD14 and TLR4 (Fig 3B) were significantly higher in liver tissue of TG mice than in WT mice after 24-week ethanol intake.

### NFκB and IL6 activation by ethanol in liver of HNP-1 transgenic mice

**Fig 4:**
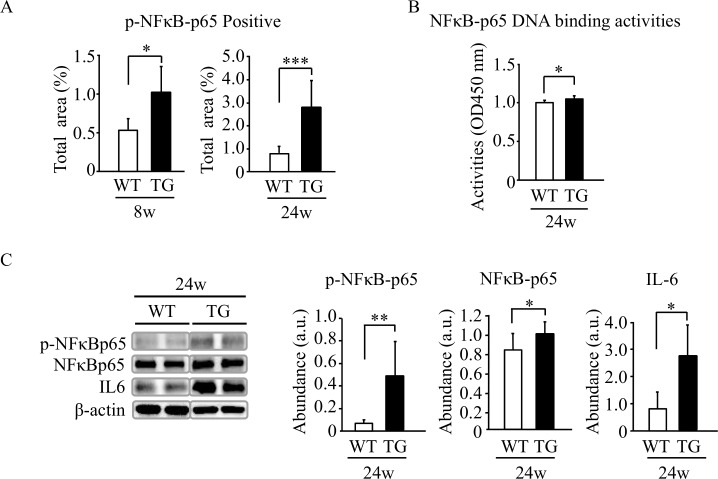
NFκB and IL-6 expression in liver tissue. (A) Semi-quantification of positive cells for immunostaining using anti-NFκB-p65 antibody in the 8- and 24-week model. (B) Semi-quantification of the nuclear NFκB-p65 activities in liver tissue. (C) Protein levels of NFκB-p65 and interleukin-6 in liver tissue assessed by Western blot analysis. Results are shown as means ± SD (7 TG mice and 6 WT mice in the 8-week intake group, and 11 TG mice and 6 WT mice in the 24-week intake group). *P<0.*05*, **P<0.*01*, ***P<0.*001*.

Since activation of hepatic stellate cells and infiltration of Kupffer cells were found in liver tissue of TG mice, NFκB and IL6 were examined to determine if NFκB activation downstream of TLR4 produced inflammatory cytokines [27,28]. The number of NFκBp-65-positive cells in liver tissue after 24-week ethanol intake was significantly greater in TG mice than in WT mice (Fig 4A and S5 Fig). NFκB-p65 DNA binding activities were significantly greater (Fig 4B) and protein levels of p-NFκB-p65, NFκB-p65 and IL6 were significantly higher (Fig 4C) in liver tissue of TG mice after 24-week ethanol intake compared to those of WT mice.

### Hepatic apoptosis is exacerbated by ethanol in HNP-1 Tg mice

**Fig 5:**
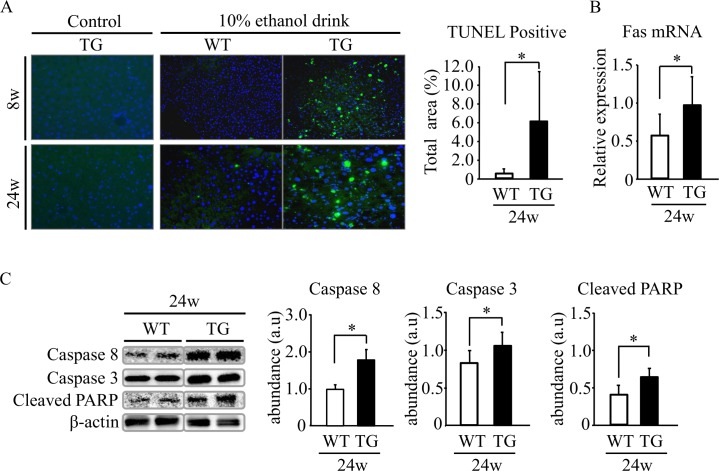
Evaluation of apoptosis in liver tissue. (A) Evaluated by TUNEL staining. (B) Relative expression of Fas mRNA in liver tissue in the 24-week model. (C) Protein levels of caspase 8 and caspase 3, and cleaved-PARP evaluated by Western blot analysis in liver tissue in the 24-week model. Results are shown as means ± SD (*n* = 11 in TG mice and *n* = 6 in WT mice).

**Fig 6:**
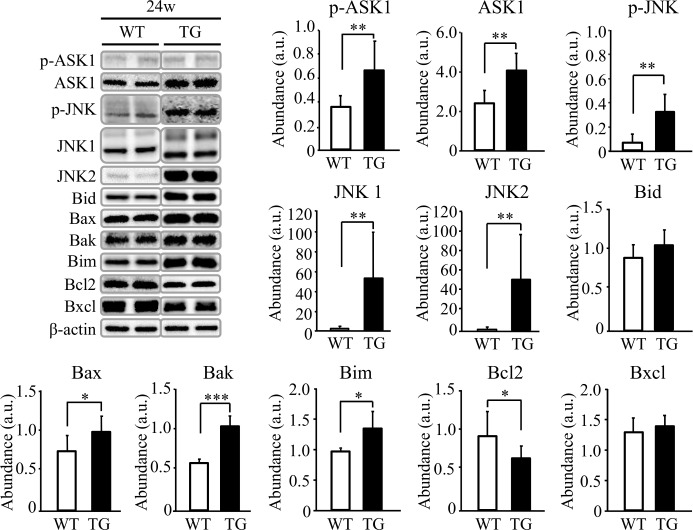
Evaluation of apoptosis-related protein using Western blot analysis in liver tissue in the 24-week model. Results are shown as means ± SD Results are shown as means ± SD (*n* = 11 in TG mice and *n* = 6 in WT mice). *P<0.*05*, **P<0.*01*, ***P<0.*001*.

Hepatocyte apoptosis was examined in TG mice because this process occurs in liver tissue of ALD patients [5,8,11,12]. Many TUNEL-positive hepatocytes were observed in liver tissue of TG mice after 8- and 24-week ethanol intake, and the number of positive cells was significantly greater in liver tissue of TG mice compared with WT mice after 24-week ethanol intake (Fig 5A). Next, we studied apoptosis-related molecules in liver tissue after 24-week ethanol intake. Fas gene expression was significantly higher in liver tissue of TG mice (Fig 5B) and expression of proteins such as caspase 8, caspase 3, and cleaved-PARP was also greater in liver tissue of TG mice (Fig 5C). In addition, p-ASK1, ASK1, p-JNK, JNK1, JNK2, Bax, Bak and Bim expression levels were significantly higher in liver tissue of TG mice after 24-week ethanol intake (Fig 6), and Bcl2 expression was significantly inhibited in these mice (Fig 6). Hepatic expression of Bcl-2 mRNA after 24-week ethanol intake was also lower in TG mice compared to WT mice (S6 Fig), although the difference was not significant (P = 0.07). These results suggest that HNP-1 might be involved in hepatocyte apoptosis.

### HNP-1 influences miRNA expression in the liver

Expression levels of 84 miRNAs in liver tissues from TG and WT mice were compared after 24-week ethanol intake, and six miRNAs were found to be significantly overexpressed in livers from TG mice compared to WT mice (S3 Table). Among these miRNAs, miRNA-34a-5p showed the largest significant difference (S3 Table).

### ER stress is enhanced by HNP-1

The level of catalase, an antioxidant enzyme, tended to be higher in TG mice after 8-week ethanol intake and was significantly higher in these mice after 24-week ethanol intake, compared to WT mice (S7 Fig). The protein level of CHOP was also significantly higher and that of PUMA showed a tendency to be higher in liver tissue of TG mice after 24-week ethanol intake, suggesting that ER stress was increased (S7 Fig).

### Apoptosis of SK-Hep1 induced by ethanol was enhanced by HNP-1

**Fig 7:**
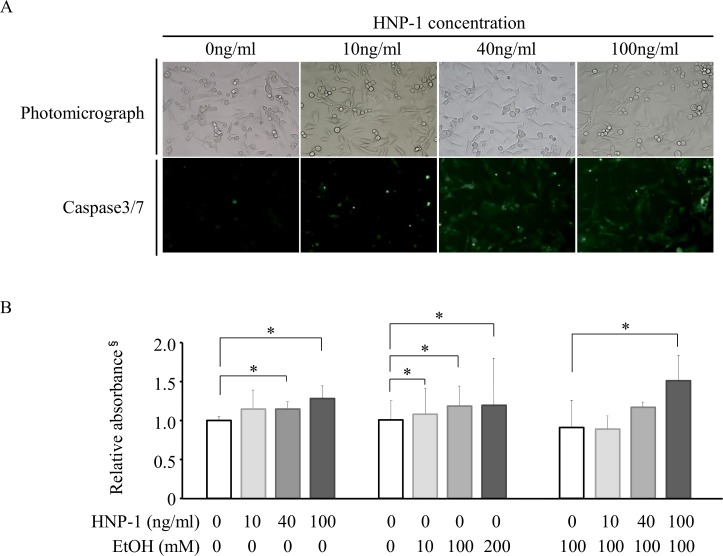
Apoptosis in human hepatic adenocarcinoma cell line SK-Hep-1 by ethanol and HNP-1 assessed by caspase 3/7 activity and DNA fragmentation. (A) Activity of caspase 3/7. (B) DNA fragmentation. *P<0.*05*, ^§^A405 nm-A480 nm. Results are shown as means ± SD (*n* = 4 for each group).

**Fig 8:**
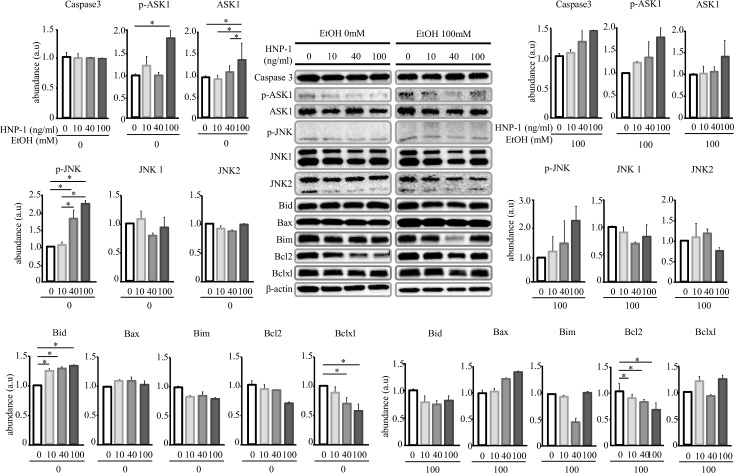
Relative expression of apoptosis-related protein in human hepatic adenocarcinoma cell line SK-Hep-1 assessed by Western blot analysis. Results are shown as means ± SD (*n* = 4 for each group). *P<0.*001*.

**Fig 9:**
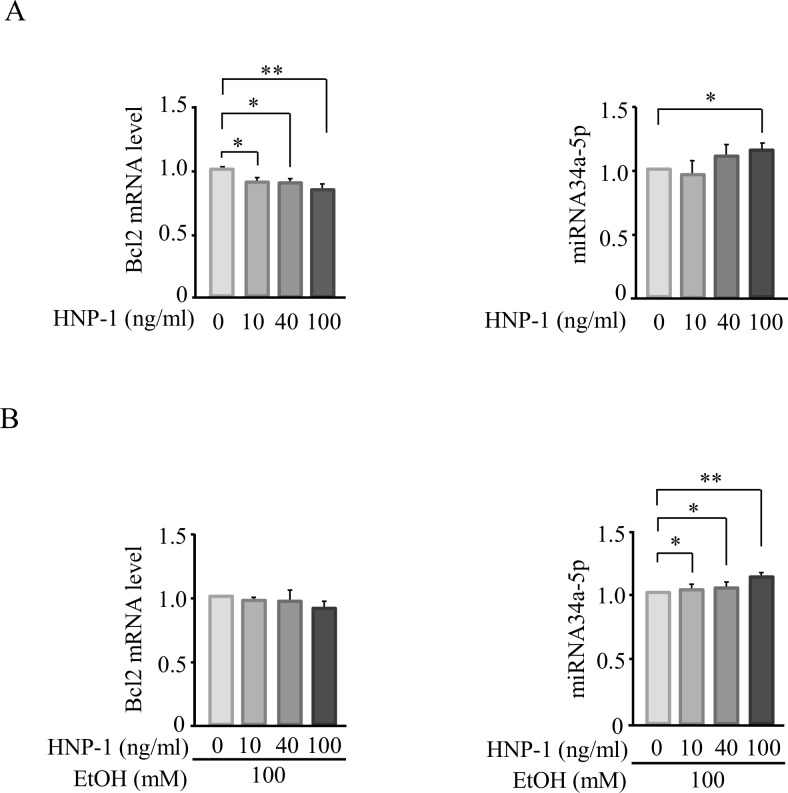
Expression of mRNA levels of Bcl2 and microRNA 34a-5p in the human hepatic adenocarcinoma cell line SK-Hep-1. (A) In the absence of ethanol. (B) In the presence of 100 mM ethanol. Results are shown as means ± SD (n = 4 for each group). *P<0.*05*, **P<0.*01*.

HNP-1 increased caspase 3/7 activity in SK-Hep1 cells in a concentration-dependent manner and promoted apoptosis of these cells (Fig 7A and 7B). Similarly, in the presence of 100 mM ethanol, HNP-1 further promoted apoptosis of SK-Hep1 cells (Fig 7B). In the presence of 100 mM ethanol, an analysis of apoptosis-related protein expression showed that HNP-1 significantly decreased Bcl2 expression in a concentration-dependent manner (Fig 8). HNP-1 also tended to increase expression levels of caspase 3, p-ASK1, ASK1, p-JNK, and Bax in SK-Hep1 cells in the presence of ethanol (Fig 8). In addition, HNP-1 decreased mRNA levels of Bcl2 and increased miRNA34a-5p expression in SK-Hep1 cells in the absence of ethanol (Fig 9A), and these effects were similar in the presence of 100 mM ethanol (Fig 9B).

## Discussion

Neutrophil infiltration in the liver is a prominent feature of ALD, and is related to the severity of the disease [4,5,11,12,20]. In addition, our preliminary data suggest that serum levels of HNP-1 in patients with alcoholic liver disease are higher than those in healthy controls (data not shown). Under inflammatory conditions, HNPs induce production of cytokines and chemokines, which may contribute to progression of ALD [11–14,20]. However, the role of neutrophils and HNP-1 in ALD is still unknown. Thus, we have established a strain of TG mice expressing HNP-1 cDNA under the control of a β-actin-based CAG promoter [23]. In the current study, we examined the effects of HNP-1 on ALD pathogenesis in an ethanol-induced mouse ALD model. HNP-1 did not influence the degree of hepatic steatosis, but increased Kupffer cell infiltration in liver and promoted hepatocyte apoptosis, accompanied by increased hepatic fibrosis.

In this study, liver weight of TG mice was increased compared with that of WT mice after 24-week ethanol intake. We speculated that the increased liver weight in TG mice was due to increased hepatic steatosis. However, we also found that HNP-1 did not influence hepatic steatosis caused by ethanol. Hepatic steatosis is an initial lesion in alcoholic liver injury and occurs by deposition of triglycerides, phospholipids, and cholesteryl esters in hepatocytes, which suggests that hepatic steatosis may be directly or indirectly associated with hepatic expression of lipogenic genes. Genes such as SREBP-1, ACC and SCD1 showed no differences in expression levels between TG and WT mice after 24-week ethanol intake (data not shown). These findings suggest that HNP-1 has no effect on these lipogenic genes, and the mechanism of increased liver weight in TG mice remains undetermined.

Hepatocyte apoptosis is an important pathologic feature of ALD [4,5,8,11–14]. Apoptotic hepatocytes often colocalize with infiltrating neutrophils, suggesting an inflammatory response triggered by apoptosis [4,5,8,11–14]. HNP-1 induces cancer cell apoptosis directly [29] and intracellular expression of HNP-1 induces apoptosis that inhibits tumor growth [30,31]. HNP-1 also increases inhibition of proliferation and apoptosis in the 4T1 breast cancer model mouse and enhances mitochondrial damage and apoptosis in 4T1 cells [32]. Chemokine release and HNP-1-induced inflammation and apoptosis occur in human bronchial and alveolar epithelial cells [33]. These reports suggest that HNP-1 has concentration-dependent pro-inflammatory and apoptotic effects *in vitro* and *in vivo*. The current study suggests that HNP-1 induces similar apoptotic effects in ALD.

Chronic alcohol consumption promotes small intestinal permeability and allows invasion of the liver by lipopolysaccharide (LPS) through the portal vein [27,34]. LPS binds to LPS-binding protein (LBP) and activates hepatic Kupffer cells via CD14, Mac-1, and TLR-4 [27,28,34]. The activated Kupffer cells produce inflammatory cytokines, such as IL-1, IL-6, IL-8 and TNF-α, and ROS [27,28,34]. LPS also stimulates mesenchymal cells in bone marrow to produce granulocyte-colony stimulating factor (G-CSF), which promotes production of bone marrow neutrophils [35]. Neutrophils migrate to inflamed sites, and thus onset of ALD in TLR4-knockout mice is inhibited and ethanol-induced liver injury is relieved in CD14- or LBP-knockout mice [36,37]. The current study showed that expression of CD14 and TLR4 in liver tissue after 24-week ethanol intake was significantly higher in TG mice compared with WT mice, and that Kupffer cells significantly increased in TG mice, which suggests that the activated Kupffer cells might produce inflammatory cytokines and ROS. Catalase expression was also significantly greater in liver of TG mice after 24-week ethanol intake, which suggests that excessive generation of ROS induces strong oxidative stress. Kupffer cells and neutrophils invade the liver and produce ROS [11–14,27,34,38], and HNP-1 secreted by neutrophils acts as a chemotactic factor in macrophages and T lymphocytes [39]. Our findings suggest that promotion of Kupffer cell migration and infiltration is followed by production of chemokines, inflammatory cytokines, and ROS by the activated Kupffer cells, which then lead to hepatocellular damage.

TNF family members trigger apoptosis through high Bax expression via caspase 8 from Fas and through a pathway in which JNK is activated via ASK1 from Fas to produce high Bax expression. Apoptosis is also enhanced by inhibition of Bcl2, an anti-apoptotic protein. HNP-1 induced apoptosis of SK-Hep1 cells via caspase 3/7 and more strongly induced apoptosis in the presence of ethanol *in vitro*, while significantly decreasing Bcl2 expression. All these effects occurred in a concentration-dependent manner. These findings suggest that HNP-1 directly inhibits Bcl2 expression to promote hepatocyte apoptosis.

After 24-week ethanol intake, miRNA-34a-5p was significantly upregulated in liver tissue of TG mice compared to WT mice. Members of the miRNA-34a family are mediators of the p53 tumor suppressor gene, and p53-dependent upregulation induces cell cycle arrest and apoptosis [40,41]. miRNA-34a directly inhibits Bcl2 expression, and miRNA-34a expression leads to G1 cell cycle arrest and Bcl2 downregulation [40,41]. In addition, Bcl2 expression is inhibited in miRNA-34a upregulated transgenic mice [42]. High miRNA-34a expression has also been observed in a chronic ethanol-fed rat model and in liver tissue of patients with alcoholic liver injury [43]. Strong overexpression of miRNA-34a was found in liver tissue of ethanol-fed mice and in ethanol-treated N-Heps, HiBECs, and HepG2 cells [44]. In addition, HNP-1 affected expression of miRNA-34a-5p *in vitro* in SK-Hep1 cells with a decrease in Bcl2 expression, although the change of Bcl2 mRNA expression was not significant. In contrast, HNP-1 did not induce significant apoptosis in ethanol-treated HepG2 cells (data not shown). Cell specificity, concentrations of HNP-1 and ethanol, and time courses of expression of protein and mRNA require further study. On the other hand, expression of proteins including CHOP increased in TG mice after 24-week ethanol intake, indicating elevated ER stress, although we did not examine an association between HNP-1 and ER stress *in vitro*. Bax activation and translocation to mitochondria promotes downstream CHOP signaling [45], with a proposed mechanism of ER stress-induced apoptosis as the intrinsic pathway. PUMA expression also tended to be higher in TG mice after 24-week ethanol intake. PUMA activates pro-apoptotic proteins, Bax and Bak, releases cytochrome C, and inhibits Bcl2 expression [46]. Thus, HNP-1 enhances miRNA-34a expression in the presence of ethanol or induces ER stress, which might result in inhibition of Bcl2 expression and promotion of apoptosis.

We previously showed that HNP-1 directly promotes proliferation of LI90 hepatic stellate cells [23] and promotes liver fibrosis in a mouse model of NAFLD. In addition, HNP-1 concentration-dependently activated LI90 cells in the absence and presence of ethanol (data not shown), and neither induced apoptosis of LI90 cells nor affected protein expression of Bax and Bcl2 in these cells (data not shown). These results suggest that HNP-1 might directly activate hepatic stellate cells and promote liver fibrosis, but not affect apoptosis in stellate cells. In contrast, hepatocyte apoptosis is significantly increased in patients with alcoholic hepatitis, and the disease severity is correlated with liver fibrosis [4,5]. Studies in animal models show progression of liver fibrosis after increased hepatocyte apoptosis and activation of hepatic stellate cells by inflammation and chemokine production [47]. Furthermore, apoptotic bodies due to hepatocyte apoptosis are phagocytozed by Kupffer cells and hepatic stellate cells, which trigger activation of hepatic stellate cells, enhanced expression of genes such as TGF-β, and progression of hepatic fibrosis [47]. These findings suggest the presence of a mechanism in which onset of hepatocyte apoptosis induces liver fibrosis in ALD. Therefore, HNP-1 in ALD may enhance liver fibrosis via both hepatocyte apoptosis and hepatic stellate cells activation, although further studies in primary hepatocytes and stellate cells are needed.

Treatment for severe alcoholic hepatitis includes administration of corticosteroids, pentoxifylline, anti-TNF-α antibody, and plasma exchange and hemodialysis, but the efficacy is unclear [4,5]. The plasma concentrations of inflammatory cytokines such as TNF-α, IL-1, IL-6 and IL-8 are high in patients with alcoholic hepatitis [4,48]. White blood cells, and in particular neutrophils, are increased by inflammatory reactions [20,21,35]. The significance of hepatocyte apoptosis has been shown in alcoholic liver injury models [13,47,49]. Hepatocyte apoptosis colocalizes with neutrophil infiltration, and thus it is likely that apoptosis triggers inflammation [4,5,8,11–14]. Therefore, leukapheresis may be an effective treatment for alcoholic hepatitis[21,48]. The current results suggest that HNP-1 might promote liver fibrosis and hepatocyte apoptosis in progression of ALD. HNP-1 strongly inhibits spontaneous apoptosis of human neutrophils [50], and increases macrophages, azurophilic granules, and T lymphocytes in bacteria-infected mice, in which induced HNP-1 accumulation is accompanied by increased leukocyte accumulation in the infected sites [51]. In alcoholic liver injury, prolongation of the neutrophil lifespan by HNP-1 might cause persistence of HNP-1 effects and aggravation of the pathology. Therefore, removal of HNP-1 may be effective for inhibition of progression of ALD.

We used the CAG promoter to drive HNP-1 expression. This promoter results in nonspecific gene expression *in vivo* [23]. However, HNP-1 probably circulates through the human body, including in liver tissues. α-Defensins including HNP-1 become biologically active after synthesis, processing into mature peptides, and extracellular release [52]. These observations suggest that the precise cell types expressing HNP-1 may not markedly affect the results of our experiments.

## Conclusion

Onset of ALD induces LPS and oxidative stress via acetaldehyde and ROS, which activate Kupffer cells and produce inflammatory cytokines such as TNF-α and chemokines such as MCP-1. Neutrophils then migrate to the inflamed sites and infiltrate the liver. The current study shows that HNP-1 secreted upregulates miRNA-34a and inhibits Bcl2 expression, which may directly induce and promote hepatocyte apoptosis. In turn, hepatocyte apoptosis may promote liver fibrosis. The study also suggested that HNP-1 secreted by neutrophils activates hepatic stellate cells in patients with alcoholic liver injury to increase production of collagen and promote liver fibrosis. Thus, HNP-1 secreted by neutrophils is an important mediator of the progression of hepatic fibrosis and hepatocyte apoptosis in alcoholic liver disease.

## Supporting information

S1 The ARRIVE Guidelines checklist (PDF)

S1 Fig Histological findings of liver tissues. (TIF)

S2 Fig Hepatic steatosis induced by ethanol with or without HNP-1 expression. (TIF)

S3 Fig Expression of α-smooth muscle actin in liver tissue assessed by immunostaining using anti-α-smooth muscle actin antibody in liver tissue. (TIF)

S4 Fig Expression of F4/80 and CD68 in liver tissue assessed by immunostaining using anti-F4/80 or anti-CD68 antibody. (TIF)

S5 Fig NFκB expression in liver tissue in the 8- and 24-week models assessed by immunostaining using anti-NFκB-p65 antibody. (TIF)

S6 Fig Hepatic mRNA expression of Bcl2 in liver tissue in the 24-week model. (TIF)

S7 Fig Endoplasmic reticulum stress-associated protein in liver tissue in the 24-week model. (TIF)

S1 Table Real-time qRT-PCR primer sequences. (DOCX)

S2 Table Characteristics of ethanol-induced liver injury in mice. (DOCX)

S3 Table microRNA expression in liver tissue in HNP-1 transgenic mice compared to that in wild type mice under 24-weeks ethanol intake. (DOCX)
