# Supplementary figures and images for: Human neutrophil peptide-1 promotes alcohol-induced hepatic fibrosis and hepatocyte apoptosis
Source: PLoS One. 2017 Apr 12;12(4):e0174913. doi: 10.1371/journal.pone.0174913 (PMC5389644; doi:10.1371/journal.pone.0174913)

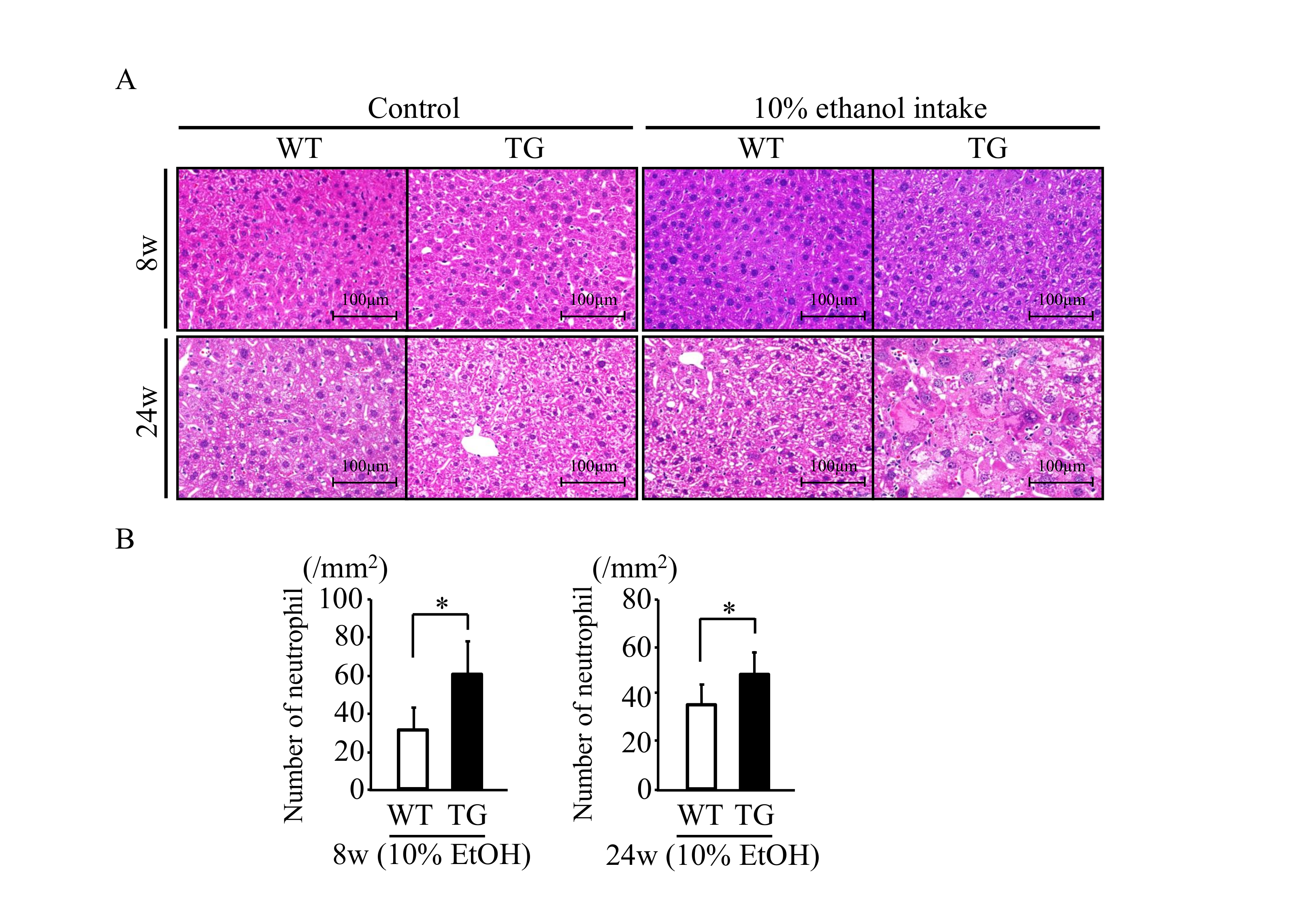

Supplement: S1 Fig — (TIF) [file pone.0174913.s002.tif]

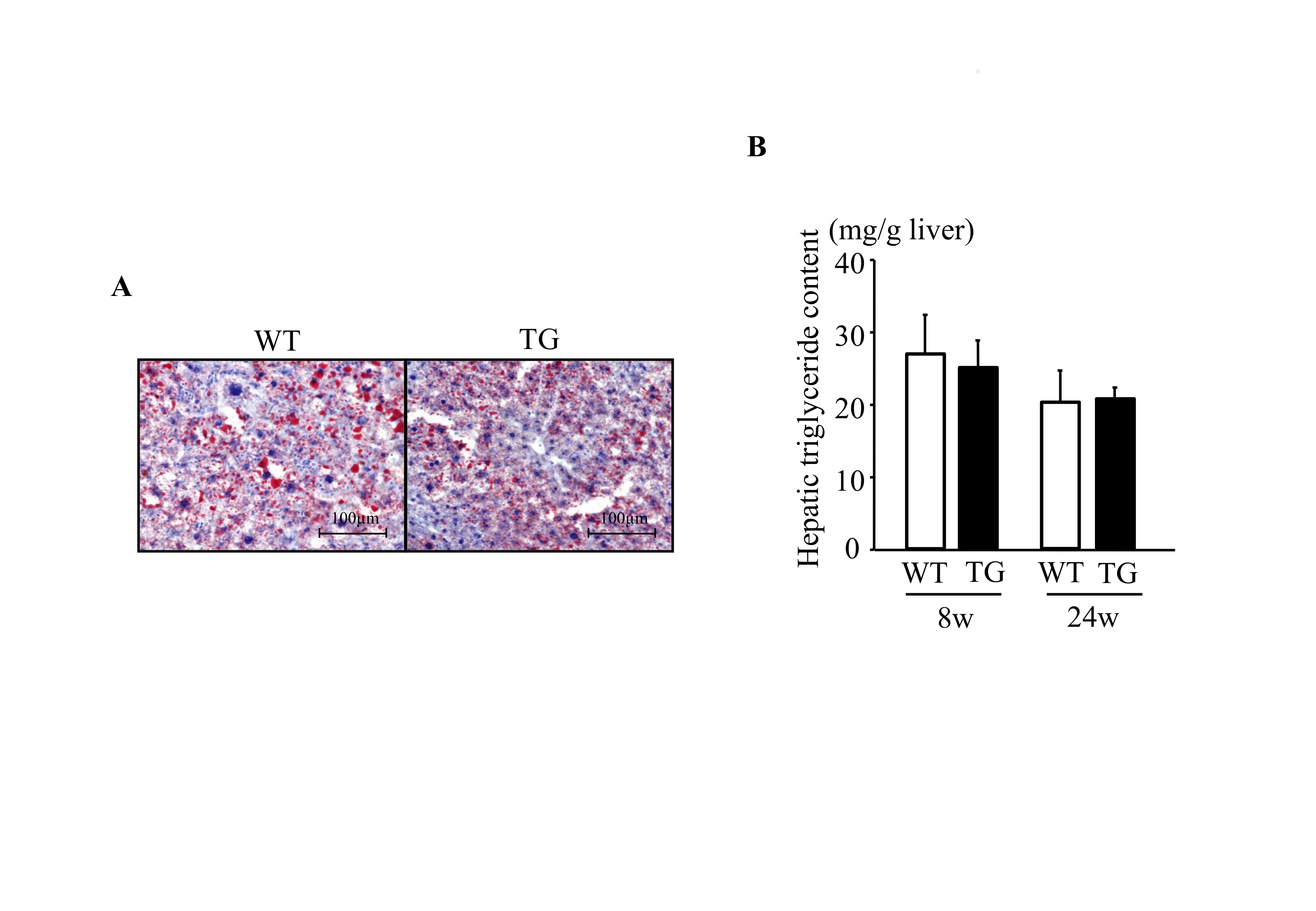

Supplement: S2 Fig — (TIF) [file pone.0174913.s003.tif]

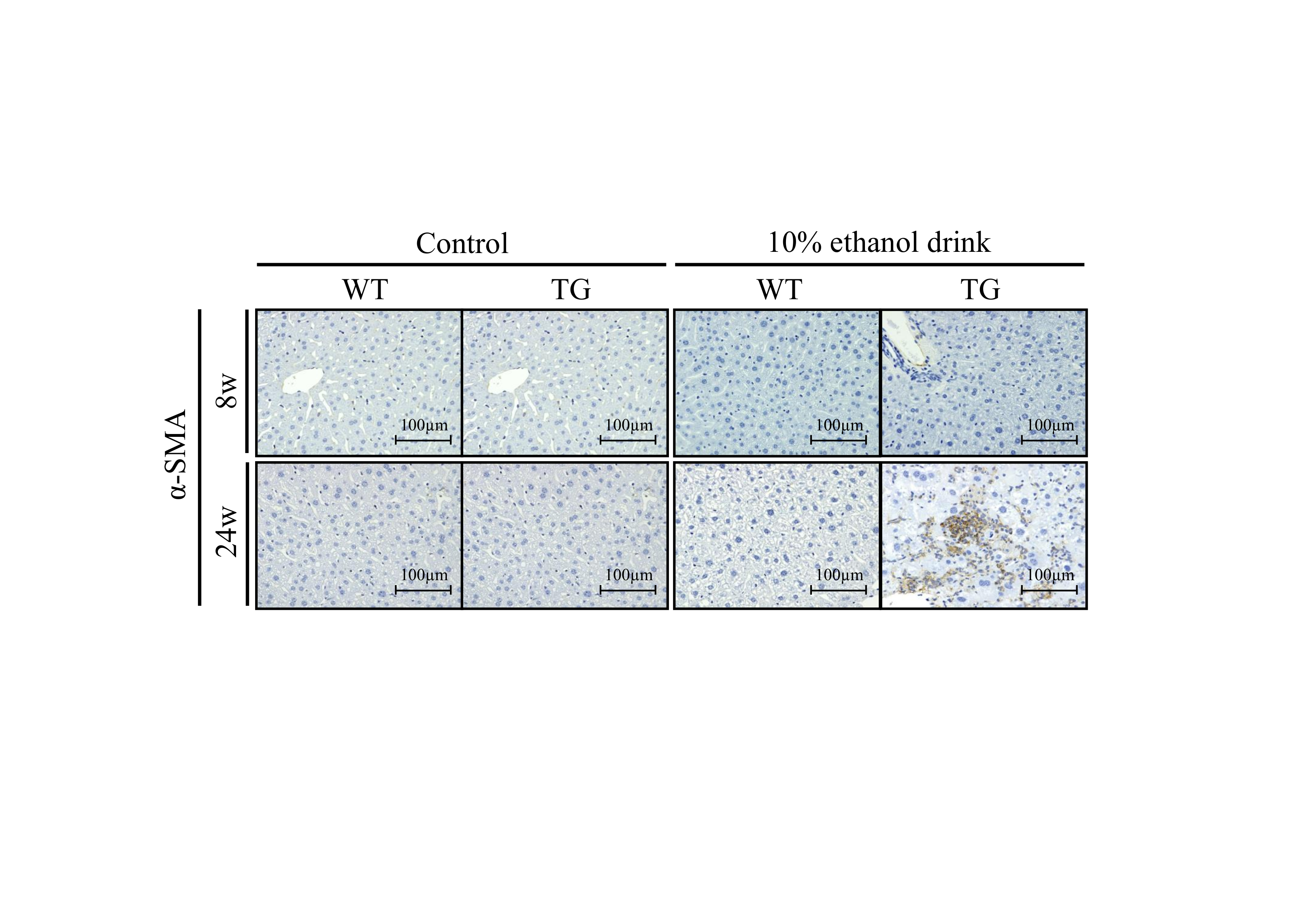

Supplement: S3 Fig — (TIF) [file pone.0174913.s004.tif]

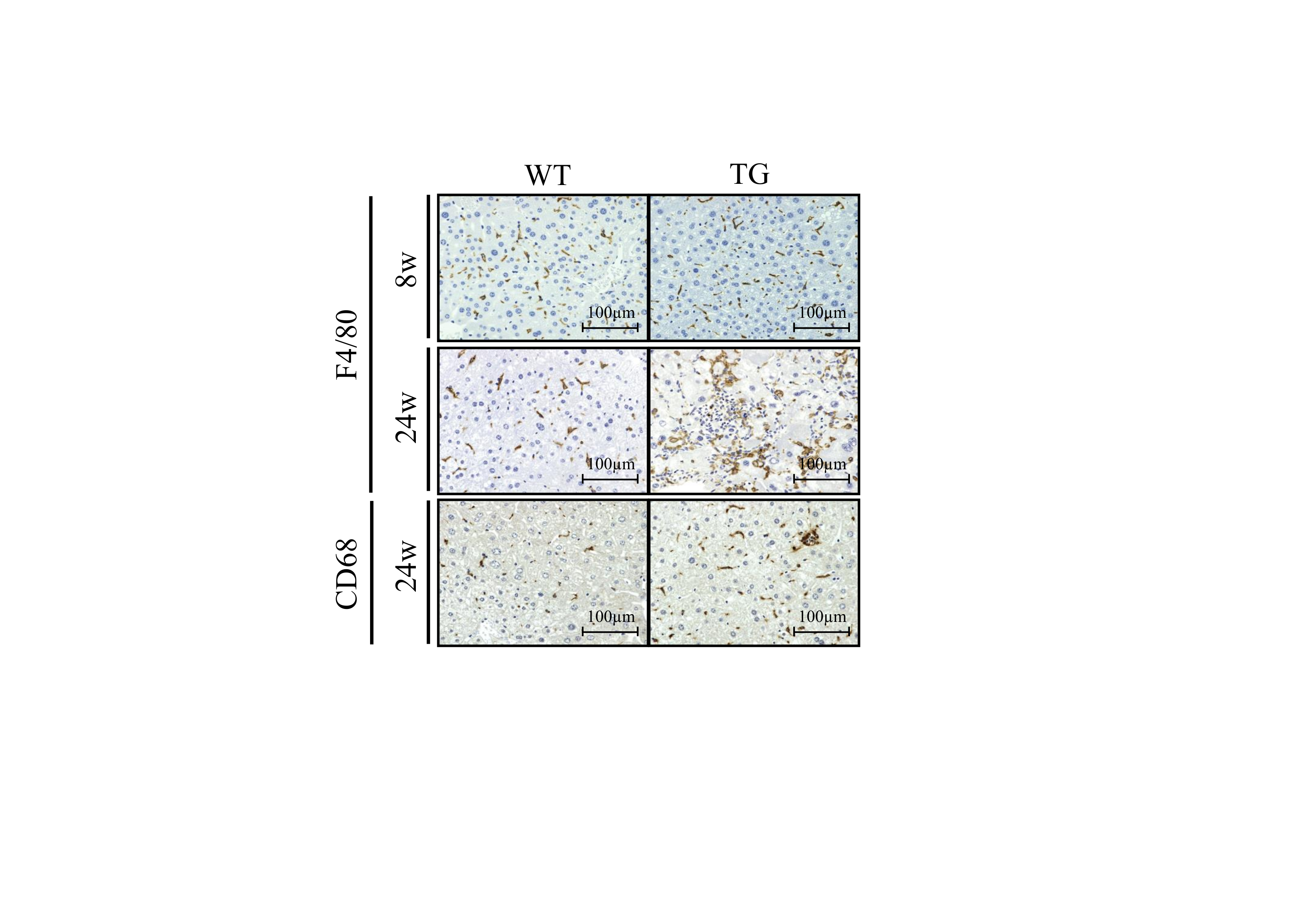

Supplement: S4 Fig — (TIF) [file pone.0174913.s005.tif]

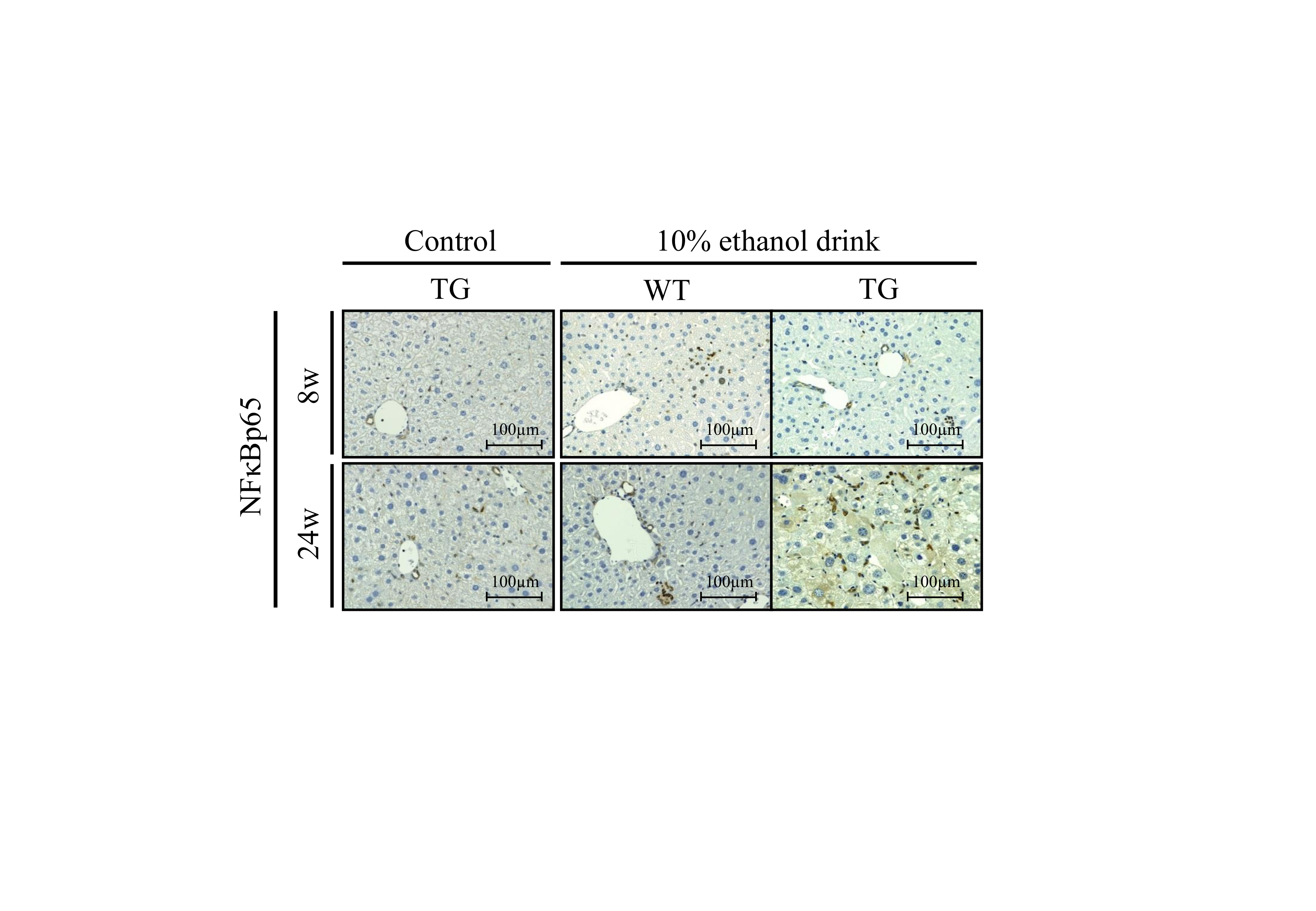

Supplement: S5 Fig — (TIF) [file pone.0174913.s006.tif]

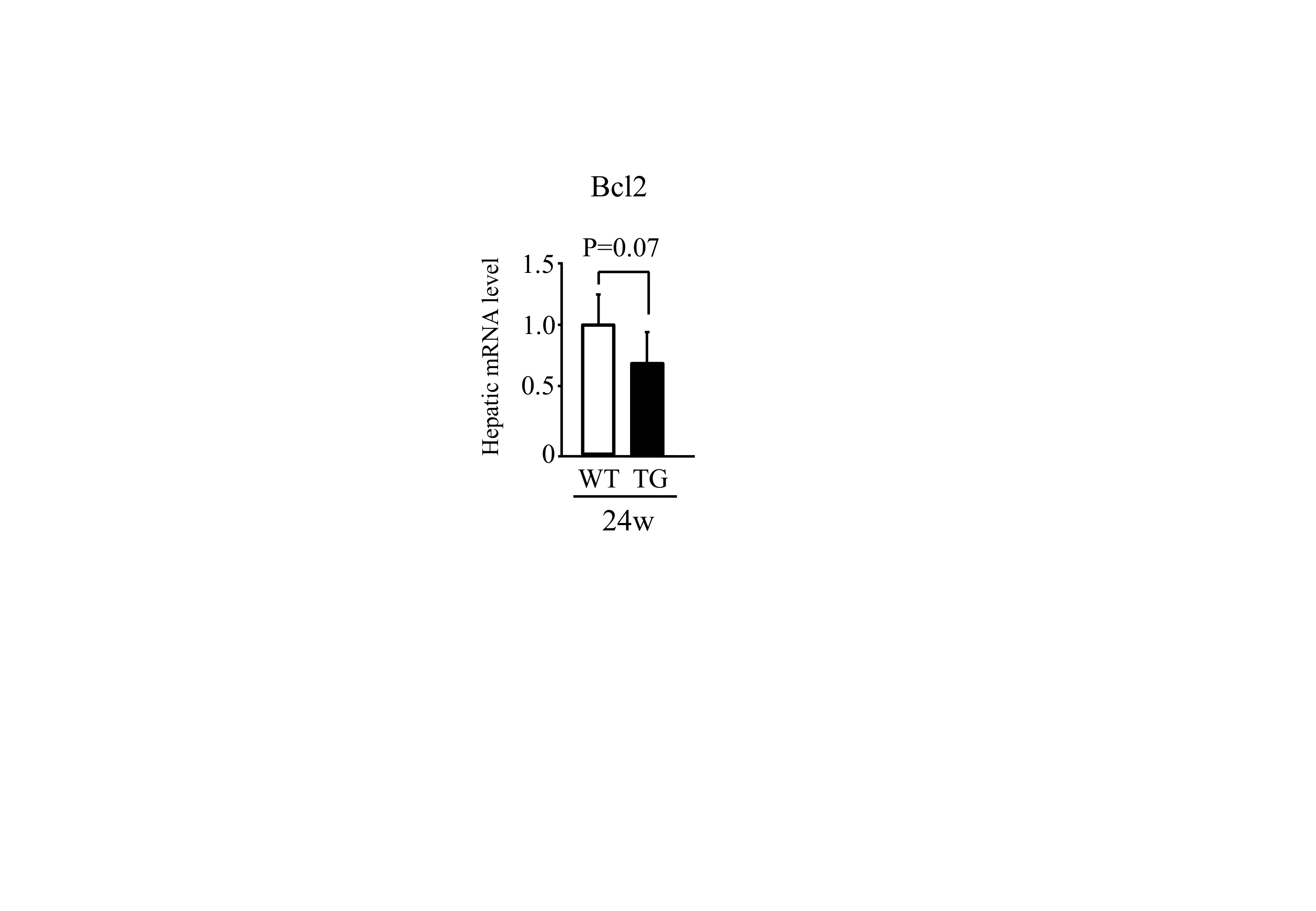

Supplement: S6 Fig — (TIF) [file pone.0174913.s007.tif]

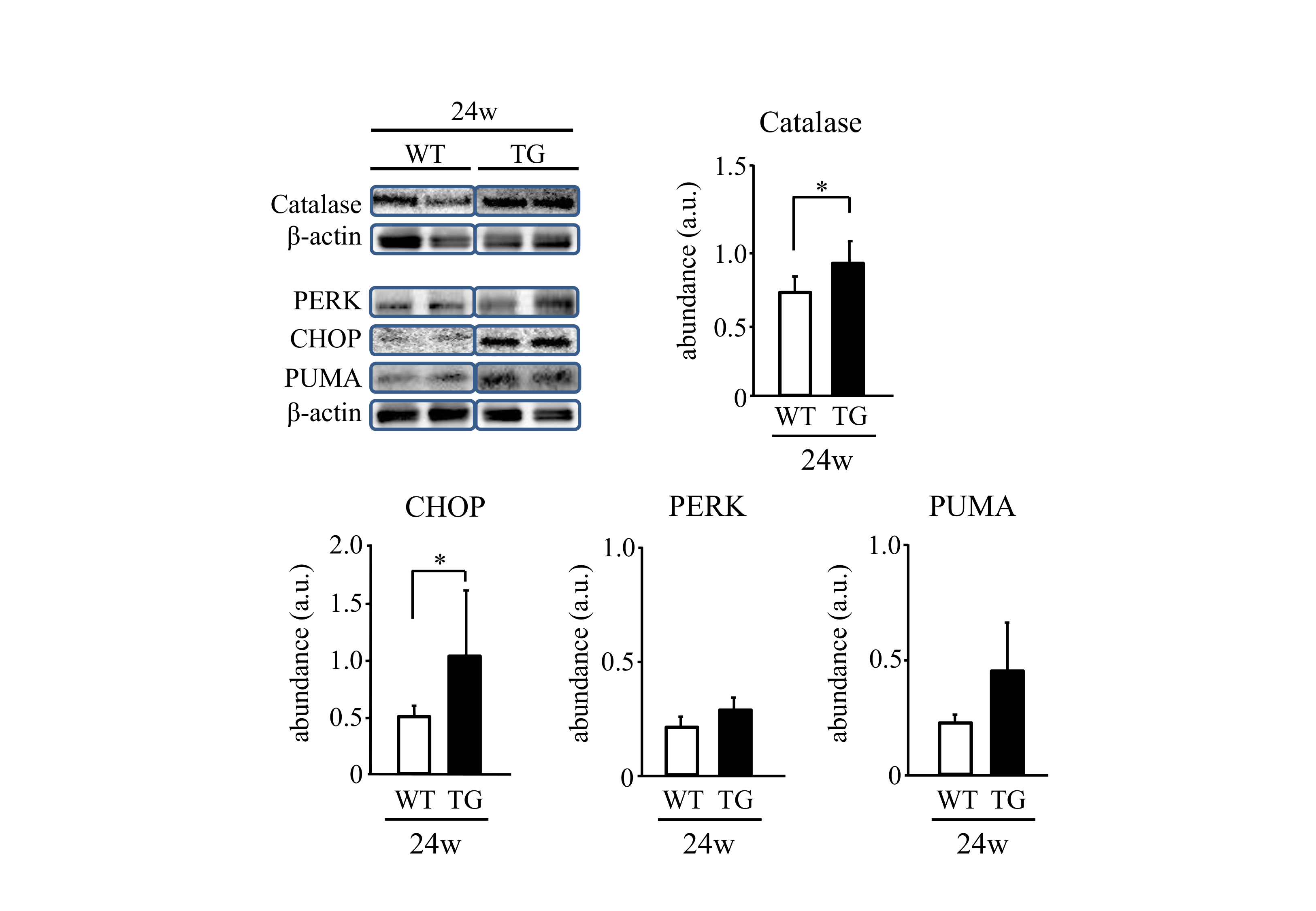

Supplement: S7 Fig — (TIF) [file pone.0174913.s008.tif]
