# Supplementary material for: Human neutrophil peptide-1 promotes alcohol-induced hepatic fibrosis and hepatocyte apoptosis
Source: PLoS One. 2017 Apr 12;12(4):e0174913. doi: 10.1371/journal.pone.0174913 (PMC5389644; doi:10.1371/journal.pone.0174913)
Supplement: S1 Table — (DOCX) [file pone.0174913.s009.docx]

S1 Table. Real-time qRT-PCR primer sequences.

| Gene | Forward Primer (5’→3’) | Reverse Primer (5’→3’) |
| --- | --- | --- |
| Mouse GAPDH | AAATGGTGAAGGTCGGTGTGAAC | CAACAATCTCCACTTTGCCACTG |
| Mouse collagen1a1 | GACATGTTCAGCTTTGTGGACCTC | GGGACCCTTAGGCCATTGTGTA |
| Mouse Fas | GCTGTCAACCATGCCAACC | AAGTTGCATCCACCCAAATCA |
| Mouse Bcl2 | CTTTCTGCTTTTTATTTCATGAG | CAGAAGATCATGCCGTCCTT |
| Human  GAPDH | GCACCGTCAAGGCTGAGAAC | TGGTGAAGACGCCAGTGGA |
| Human  Bcl2 | TGGACAACCATGACCTTGGAC | GTGCTCAGCTTGGTATGCAGAA |

GAPDH, Glyceraldehyde 3-phosphate dehydrogenase.
