# Supplementary material for: Human neutrophil peptide-1 promotes alcohol-induced hepatic fibrosis and hepatocyte apoptosis
Source: PLoS One. 2017 Apr 12;12(4):e0174913. doi: 10.1371/journal.pone.0174913 (PMC5389644; doi:10.1371/journal.pone.0174913)
Supplement: S2 Table — (DOCX) [file pone.0174913.s010.docx]

S2 Table. Characteristics of ethanol-induced liver injury in mice.

|  | 24w WT | 24w TG | *P*　value* |
| --- | --- | --- | --- |
| BW(g) | 33.4±2.3 | 34.2±2.7 | 0.448 |
| Liver weight (g) | 1.7±0.1 | 2.0±0.3 | 0.004 |
| Liver/Weight (%) | 5.0±0.2 | 5.8±0.5 | 0.138 |
| ALT (IU/l) | 35.8±6.9 | 78±81 | 0.037 |
| Glucose (mg/dl) | 162.7±14.1 | 184.3±37.6 | 0.120 |
| Triglyceride (mg/dl) | 72.2±35.3 | 66.5±33.2 | 0.727 |
| Total cholesterol (mg/dl) | 80±36.2 | 94.5±30.9 | 0.901 |

Values are shown as mean ± SD; WT, wild type; TG, HNP-1 transgenic mice; BW, body weight; *evaluated by Mann-whitney test.
