# Supplementary material for: Human neutrophil peptide-1 promotes alcohol-induced hepatic fibrosis and hepatocyte apoptosis
Source: PLoS One. 2017 Apr 12;12(4):e0174913. doi: 10.1371/journal.pone.0174913 (PMC5389644; doi:10.1371/journal.pone.0174913)
Supplement: S3 Table — (DOCX) [file pone.0174913.s011.docx]

S3 Table. microRNA expression in liver tissue in HNP-1 transgenic mice compared to that in wild type mice under 24-weeks ethanol intake.

| miRNA | Fold change | P value |
| --- | --- | --- |
| miR-7a-5p | 1.505 | 0.045 |
| miR-15b-5p | 1.462 | 0.021 |
| miR-27a-3p | 1.388 | 0.014 |
| miR-34a-5p | 2.155 | 0.005 |
| miR-125a-5p | 1.816 | 0.048 |
| miR-183-5p | 1.805 | 0.039 |
